# Early detection of colon cancer by amino acid profiling using AminoIndex Technology: a case report

**DOI:** 10.1186/1746-1596-8-203

**Published:** 2013-12-10

**Authors:** Junichi Yatabe, Midori Sasaki Yatabe, Katsuyuki Ishibashi, Yoshihiro Nozawa, Hironobu Sanada

**Affiliations:** 1 Department of Nephrology, Hypertension, Diabetology, Endocrinology and Metabolism, Fukushima Medical University School of Medicine, Fukushima, Japan; 2 Department of Pharmacology, Fukushima Medical University School of Medicine, Fukushima, Japan; 3 Department of Gastrointestinal Medicine, Bange Kosei General Hospital, Fukushima Welfare Federation of Agricultural Cooperatives, Fukushima, Japan; 4 Department of Pathology, Shirakawa Kosei General Hospital, Fukushima Welfare Federation of Agricultural Cooperatives, Fukushima, Japan; 5 Department of Tumor and Host Bioscience, Fukushima Medical University School of Medicine, Fukushima, Japan; 6 Division of Health Science Research, Fukushima Welfare Federation of Agricultural Cooperatives, Fukushima, Japan

**Keywords:** Plasma amino acid, Colorectal cancer, Adenoma, AminoIndex Cancer Screening

## Abstract

Most tumor markers have low detection rates for curable stages of cancer and are therefore not satisfactory for use in a healthy population. A new cancer risk calculation method, AminoIndex Cancer Screening (AICS), uses multiple plasma amino acid concentrations to calculate the risks for several cancers simultaneously and is suggested to have a high detection rate for early-stage cancers and a low false-positive rate for adenomas. Here, we describe a male patient with a family history of colorectal cancer who underwent AICS. He was judged as Rank C for colorectal cancer, which reportedly has a specificity of 95%, a sensitivity of 41%, and an estimated positive predictive value of 0.67%. He underwent colonoscopy for a secondary screening, which revealed a 10-mm adenoma-like lesion in the ascending colon, with a biopsy report of partial carcinoma. The tumor was endoscopically removed and diagnosed as carcinoma in situ (carcinoma in adenoma). This early detection method allowed complete resection of the carcinoma, and the patient is in remission. This is the first case in which a curable cancer was detected using AICS. With its high detection rate for early-stage cancers and its ease of use, this tool, which recently became available in Japan, may be beneficial for simultaneous screening of the general population for multiple cancers.

**Virtual slides:**

The virtual slide(s) for this article can be found here: http://www.diagnosticpathology.diagnomx.eu/vs/2145080259887842

## Background

Colorectal cancer is the third most common cancer in the world, and the incidence rate of colorectal cancer is increasing in Japan as diets become more westernized [1]. As with most cancers, colorectal cancer survival is highly dependent on the stage of disease at diagnosis. Typical 5-year survival rates for colorectal cancer are 90% for cancers detected at the localized stage, 70% for the regional stage, and 10% for people diagnosed with distant metastatic cancer [2]. Early detection is suggested to be beneficial; however, currently available methods such as fecal occult blood testing and colonoscopy are problematic owing to the low specificity and invasiveness, respectively. In addition, detection rates of these methods and tumor markers for early-stage cancers are not high enough. As such, an effective method for screening has not been established at present.

Here, we report a case of an early-stage colon cancer detected using AminoIndex Cancer Screening (AICS). AICS analysis compresses multidimensional information from the plasma free amino acid profile into a single score to maximize the differences between patients with cancer and the control population [3].

Of many different metabolites in plasma, amino acids are emerging as markers for various pathological conditions because they are at the metabolic hub, being synthesized and degraded in many key processes [4]. Reportedly, plasma amino acid profiling was useful in diabetes risk assessment [5] and the diagnosis of liver fibrosis [6]. Meanwhile, cross-sectional studies have documented the potential of plasma amino acid profiling for screening of lung [7], gastric [3], breast [8], colorectal [8], and prostate [3] cancers. AICS takes advantage of the recently popularized high performance liquid chromatography–electrospray mass spectrometry (HPLC–ESI–MS) methods that enable high-throughput analysis of plasma amino acids and both univariate and multivariate statistical analyses. To build AICS, the following 19 amino acids and related molecules are measured and statistically analyzed: alanine, arginine, asparagine, citrulline, glutamine, glycine, histidine, isoleucine, leucine, lysine, methionine, ornithine, phenylalanine, proline, serine, threonine, tryptophan, tyrosine, and valine. For the colorectal cancer AICS score, plasma concentrations of serine, proline, valine, methionine, isoleucine, and lysine are incorporated into the formula. The minimum and maximum AICS values are 0.0 and 10.0, respectively, and AICS values of 5.0 and 8.0 correspond to specificities of 80% and 95%, respectively, for each cancer [9]. There are 3 categories of AICS values: rank A, <5.0; rank B, 5.0–8.0; and rank C, ≥8.0. Subjects in the rank C category are said to have an approximately 10-fold cancer risk. Recently, Ajinomoto Co., Inc. began to commercially provide AICS in Japan for 7 types of cancers: lung, gastric, colorectal, breast, prostate, mammary, and uterine.

A characteristic of AICS is its relatively high sensitivity for early-stage cancers. For AICS Rank C colorectal cancer, sensitivities were 55% for detecting Stage 0 cancer (carcinoma in situ), 30% for Stage I cancer, 43% for Stage II cancer, 42% for Stage III cancer, and 62% for Stage IV cancer [9]. Therefore, AICS may be a good method in a healthy population, particularly because it can be easily performed and can simultaneously screen for various cancers.

## Case presentation

We report the first case of colon cancer detected using this new technology. In September 2011, a 62-year-old Japanese man whose mother died of colorectal cancer a few years before requested cancer screening. He heard about AICS, which had recently become available in Japan, and requested it because it can be performed along with routine blood testing and can simultaneously screen for multiple types of cancers.

The patient had a history of urolithiasis, intervertebral disc displacement, impaired glucose tolerance, and hypertension, for which he was receiving amlodipine at 5 mg/day. He had no history of cancer, and his annual colorectal cancer screening using fecal occult blood was negative in 2010. For his chronic lumbago and a vertebral lesion observed on magnetic resonance imaging, an orthopedic doctor recommended positron emission tomography–computed tomography (PET–CT) using 18 F-fluorodeoxyglucose in September 2011. No abnormal accumulation was observed on PET–CT (data not shown).

**Figure 1:**
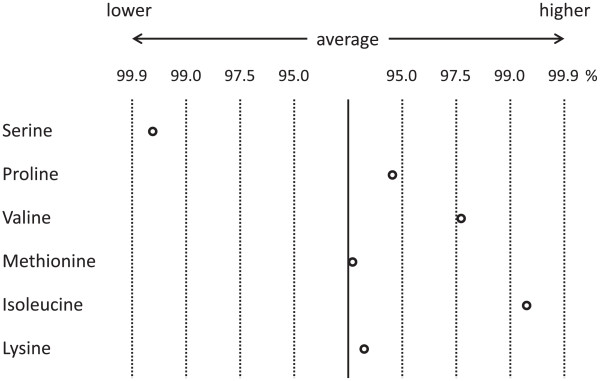
**Plasma levels of 6 amino acids used in the colorectal cancer risk calculation.** Averages and percentile ranges of the normal population are indicated because some amino acids do not show normal distribution. The data of this patient are shown as open circles. High plasma level of isoleucine is a prominent characteristic of patients with colorectal cancer.

For AICS, 5 mL of blood was collected after overnight fasting from forearm veins in tubes containing ethylenediaminetetraacetic acid and kept at approximately 0°C until plasma preparation [10]. The plasma was stored at -80°C until analysis. Plasma amino acid concentrations were measured by HPLC–ESI–MS (SRL, Inc., Tokyo, Japan) after deproteinization [11]. Ajinomoto Co., Inc. (Tokyo, Japan) commercially performed the AICS calculation using plasma amino acid concentrations. The patient’s AICS score for colorectal cancer was 8.3, which belonged to Rank C. Figure 1 shows the plasma levels of 6 amino acids used in the colorectal cancer risk calculation. High plasma level of isoleucine is a prominent characteristic of patients with colorectal cancer [3,8], which was also observed in the index patient. The patient did not have liver dysfunction, chronic kidney disease, or diabetes mellitus that may affect the results of AICS. His fasting blood glucose level was 113 mg/dL (6.28 mmol/L, normal range: 70–109 mg/dL) and glycated hemoglobin (HbA1_C_) level was 5.6% (normal range: 4.6%–6.2%). His conventional tumor markers were within the normal range: carcinoembryonic antigen (CEA) was 1.2 ng/mL (normal range: below 5.0 ng/mL) and carbohydrate antigen (CA) 19–9 was 3.6 U/mL (normal range: below 37.0 U/mL). The patient’s AICS scores for other types of cancer were also normal (gastric 0.0, lung 0.8, and prostate 0.3).

**Figure 2:**
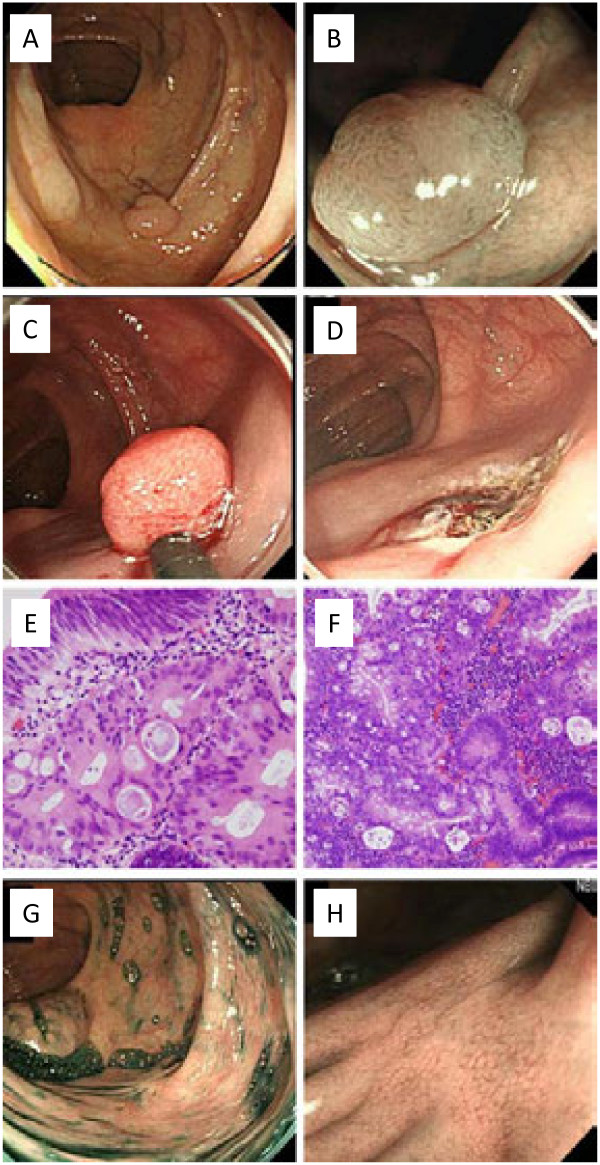
**Endoscopic and microscopic observations of the lesion. A**: the tumor observed in the ascending colon by colonoscopy, **B**: a close-up narrow-band imaging of the tumor, **C**: endoscopic submucosal dissection of the tumor, **D**: the site immediately after tumor removal, **E**: the biopsy specimen with hematoxylin–eosin staining, **F**: sections of the removed tumor with hematoxylin–eosin staining, **G**: the dissection scar, 6 months later, with indigo carmine dye, and **H**: a close-up view of the dissection scar.

AICS Rank C for colorectal cancer suggests the need for further workup such as colonoscopy. His colonoscopy in October 2011 revealed an adenoma-like lesion of 10-mm diameter in the ascending colon (Figure 2A, B). Carcinoma in adenoma was observed in the biopsy sample (Figure 2E). To remove the lesion, endoscopic mucosal resection (Figure 2C, D) was performed by a physician certified by the Japan Gastroenterological Endoscopy Society. The dissected specimen also revealed a carcinoma in situ (Figure 2F), which was the final diagnosis by an external pathology advisory board. Complete resection of the carcinoma was confirmed by the specimen, and follow-up colonoscopy after 6 months showed normal scarring, free of local recurrence (Figure 2G, H).

## Discussion

The index patient was the first case in which curable cancer was detected using AICS. In the case presented, only AICS showed the possibility of colorectal cancer at the initial screening. Fecal occult blood testing and PET–CT performed in the same year as AICS were negative. Fecal occult blood testing is commonly performed in Japan to screen for colorectal cancers. Specificity and sensitivity of fecal occult blood testing are reportedly 91.4% and 67%, respectively, for clinically significant colorectal tumors, including cancers and advanced polyps. However, its sensitivity decreases for tumors in the ascending colon, being 11.2% for cancers and adenomas of 10 mm or larger. Furthermore, these studies are limited because they analyze cancers and large adenomas together. In contrast, Rank C in AICS has a specificity of 95%, better than that reported for fecal occult blood testing, and a sensitivity of 41% for colorectal cancer. AICS also has a high detection rate for early-stage cancers, with a sensitivity of 55% for Stage 0 intramucosal carcinoma, and the sensitivity of AICS reportedly has no significant relationship with tumor staging [9]. This is in contrast to most tumor markers that have low detection rates for curable stages of cancer and are therefore not satisfactory for use in a healthy population.

In addition to its potential in detecting early disease, AICS may be more effective than fecal occult blood testing or colonoscopy for detecting cancers of the proximal colon such as the one in the index patient because plasma is used for AICS analysis. Moreover, AICS may differentiate a carcinoma from adenoma relatively well as AICS Rank C has a positive rate of 41% for colorectal cancer but only 7% for colorectal polyp. Therefore, AICS may also aid in the decision of whether biopsy should be performed in addition to general screening for an adenoma-like lesion found on colonoscopy.

It needs to be noted that AICS has only been tested for colorectal adenocarcinoma and not for other, rarer tumorous lesions in the intestines such as phyllodes tumor [12], malignant melanoma [13], and intestinal endometriosis [14]. The probable cell-type specificity is similar to other potential molecular diagnostics and prognostics methods such as special AT-rich sequence-binding protein 1 (STAB1) expression [15], beta-catenin alterations and microsatellite instability (MSI) [16] in colorectal cancer.

Also, it needs to be kept in mind that the studies on AICS have been performed only on Japanese subjects till date. Whether the same diagnostic algorithm applies to other races remains to be tested. In addition, the causality and mechanisms of plasma amino acid alterations have not been determined and are targets of further study.

## Conclusion

In summary, an early-stage colon carcinoma was found following the positive result by AICS calculated from plasma amino acid concentrations. This early detection enabled complete resection of the carcinoma. AICS could be a good method to screen the general population because it has high sensitivity for early-stage cancers and low false-positive rate for adenomas, and it can assess the risks of multiple cancers simultaneously with single blood collection.

## Consent

Written informed consent was obtained from the patient for publication of this Case Report and any accompanying images. A copy of the written consent is available for review by the Editor-in-Chief of this journal.

## Abbreviations

AICS: AminoIndex Cancer Screening; HPLC–ESI–MS: High performance liquid chromatography–electrospray mass spectrometry; PET-CT: Positron emission tomography–computed tomography.

## Competing interests

The authors declare that they have no competing interests.

## Authors’ contributions

Study concept and design were by JY and MSY. JY and KI contributed to acquisition of clinical data. Analysis and interpretation of data were performed by JY, MSY, KI, YN, and HS. JY and MSY drafted the manuscript. Administrative, technical, and material support was contributed by JY, KI, YN, and HS. JY supervised the overall study. All authors read and approved the final manuscript.
